# A decade of community-based participatory research: from a tentative start to a fruitful approach in the field of mental health: a scoping review

**DOI:** 10.1192/bjo.2021.692

**Published:** 2021-06-18

**Authors:** Aoife Janmohamed

**Affiliations:** University of Leeds Medical School

## Abstract

**Aims:**

This scoping review aimed to identify and analyse studies utilising Community-Based Participatory Research (CBPR) to design and/or disseminate a mental health (MH) intervention with underserved communities around the globe. This was with the intention of updating the knowledge base on this area, and identifying both areas of promise in this field as well as any gaps for future work to fill.

**Method:**

This scoping review was conducted using the Joanna Briggs Institute's Scoping Review Manual and the Preferred Reporting Items for Systematic reviews and Meta-Analyses extension for Scoping Reviews (PRISMA-ScR) checklist. Three databases, Scopus, PubMed and Sage Journals, were searched to identify relevant studies, using the three terms ‘CBPR’, ‘underserved’ and ‘mental health’, and words to the equivalent.

**Result:**

The search identified 607 English-language sources published between 1st January 2010 and 30th June 2020. Following duplicate removal, screening and bibliography scanning, 34 highly relevant studies remained. The studies were varied in their chosen context, MH gap, how they gave meaning to the participatory approach, how they defined their successes, and what strengths and challenges were encountered in CBPR's application to this field. Briefly, all bar one of the studies were focused on underserved communities within high-income countries (HICs); many focused solely on women and youth groups; and finally, the use of technology and talking therapies were noted to achieve particular success.

**Conclusion:**

CBPR is commonly used to engage the underserved through long-term partnership building and equitable stakeholder involvement, shifting the dialogue from research on to research with communities. This unique, needs-oriented approach harbours mutual ownership of the research, empowering historically disenfranchised individuals to become actively involved in reducing identified health disparities. In the field of MH, this is of great importance and need in many underserved communities due to issues of access, heightened by a distrust in mainstream services as well as by the stigma attached to MH conditions.

As compared to studies in this field ten years ago, CBPR has become much more established, with this review noting a remarkable increase in MH projects utilising this approach. Furthermore, the addition of new technologies to this field was shown to offer significant promise in overcoming access barriers, hoping to ultimately narrow identified MH gaps. Nonetheless, further work on the prevailing gender and HIC biases, and for a review including relevant Spanish-language studies, are still required in order to form a more global overview of this field.

